# Analysis of draft Australian rehabilitation service standards: comparison with international standards

**DOI:** 10.1186/1743-8462-5-15

**Published:** 2008-06-30

**Authors:** Susan K Graham, Ian D Cameron, Hugh G Dickson

**Affiliations:** 1 Rehabilitation Studies Unit, Royal Rehabilitation Centre Sydney, University of Sydney, Sydney, Australia; 2 Rehabilitation Unit, Liverpool Hospital, Sydney, Australia

## Abstract

**Background:**

Following her review of health systems and structures Dwyer [1] suggested that there is a need to evaluate models of care for individuals with chronic diseases. Rehabilitation services aim to optimise the activity and participation of individuals with restrictions due to both acute and chronic conditions. Assessing and optimising the standard of these services is one method of assuring the quality of service delivered to these individuals. Knowledge of baseline standards allows evaluation of the impact of health care reforms in this area of need. The aim of this article is to compare the currently available rehabilitation service standards in Australia with those used in the USA and the UK.

**Results:**

The mixed method qualitative analysis performed on the three sets of standards demonstrated repeatability and convergence via the use of triangulation. Australian Faculty of Rehabilitation Medicine (AFRM) standards were found to be consistent and concise, to provide definitions, and to cover the majority of clinically relevant issues to an extent similar to the other rehabilitation service standards. Inclusion of standards for business practices, the rehabilitation process for the person served, and outpatient and community-based rehabilitation services should be considered by the AFRM.

**Conclusion:**

The AFRM standards are an appropriate way of assessing rehabilitation services in Australia. As suggested by other workers [2,3] there should be ongoing review and field testing of the standards to maximise the relevance and utilisation of the standards.

## Background

The funding and development of rehabilitation services with the aim of reducing the impact of disability and chronic illness on population health can be difficult to plan and justify. In Australia, the Australian Council on Healthcare Standards (ACHS) EQuIP (Evaluation and Quality Improvement Programme) standards [4] are widely used for the accreditation of medical facilities. They were developed in 1996 and are up-dated on a regular basis. In 1995 the Australian Faculty of Rehabilitation Medicine (AFRM) developed rehabilitation service standards. An update of these standards [5], incorporating the EQUIP standards was in progress at the time this study was being carried out.

The American-based Commission on Accreditation of Rehabilitation Facilities (CARF) [6] has developed rehabilitation specific standards over a number of years. These are widely used by facilities in America and Sweden.

In 2000, Turner-Stokes et al [7] developed and published specialist consensus clinical standards for in-patient specialist rehabilitation in the UK for the British Society of Rehabilitation Medicine (BSRM). In 2001, the same group developed and assessed community rehabilitation service standards using a similar methodology [8]. These standards were felt to be appropriate by the majority of UK based rehabilitation specialists and have been used in quality assurance projects in UK based hospitals [9]. The two sets of standards were recently consolidated in a single set of rehabilitation service standards published by the BSRM [10].

Australian rehabilitation service standards should be useful for the purposes of rehabilitation health policy, quality assurance and research. This qualitative analysis therefore assessed the three rehabilitation specific service standards (AFRM + EQUIP- Oct 2003 draft version, BSRM and CARF) available at the time of the study. The aim of the analysis was to compare the AFRM rehabilitation service standards with standards available in the UK and USA and comment on ways in which the standards may be improved.

## Results

### Descriptive analysis

The AFRM, BSRM and EQUIP standards were developed via an interdisciplinary consultative process. The BSRM standards were also validated through the use of a questionnaire based survey, published in a peer-reviewed journal. It has not been possible to obtain details of the development and validation of the commercially developed CARF guidelines.

The AFRM and BSRM standards were developed to be advisory in nature for use in the setting up and quality assurance of services, whereas the CARF and EQUIP standards were developed specifically for the purposes of accreditation. The CARF and EQUIP standards are long-standing and have been regularly up-dated and widely used. The AFRM and BSRM standards were developed more recently and have been subject to fewer reviews.

Table 1 gives a summary of the descriptive analysis

**Table 1:** Descriptive analysis.

	**AFRM**	**BSRM**	**CARF**
**Development**	Interdisciplinary consultative via AFRM Special Projects Committee	Rehabilitation specialist consultative process	Commercially developed.
**Validation**	No formal validation	Published in peer-reviewed journals	Discussion papers
**Intent and meanings**	Advisory	Guidelines and audit of rehabilitation services in the UK	Accreditation of private rehabilitation services
**Evolution**	One update in progress since inception in 1995	Up-dated twice since developed in 2000	Up-dated annually since 1980s
**Consequences**	Limited utilisation	Voluntary utilisation in quality assurance	Accreditation actively sought

### Thematic analysis

The AFRM standards provide definitions, have consistent structure and are concise, though may not be considered comprehensive and provide no guidelines on the evaluation of services. The BSRM standards are concise with a consistent structure but no definitions or suggested service evaluation methods. The CARF standards provide definitions and an explicit evaluation technique and are comprehensive but not concise or consistent in structure. The EQUIP standards provide definitions and an explicit evaluation method. They are also concise and consistent in structure. However, they may not be considered comprehensive as they do not provide rehabilitation specific guidelines. The thematic analysis is summarised in Table 2.

**Table 2:** Thematic analysis.

	**AFRM**	**BSRM**	**CARF**
**Definitions provided**	Yes	No	Yes
**Explicit evaluation method**	No	No	Yes
**Consistent structure**	Yes	Yes	No
**Comprehensive**	No	No	Yes
**Concise**	Yes	Yes	No

### Content analysis

Table 3 provides details of the rating of the content of the three standards, and Box 1 provides a summary of the extent to which issues were covered by the AFRM standards.

**Figure 1:**
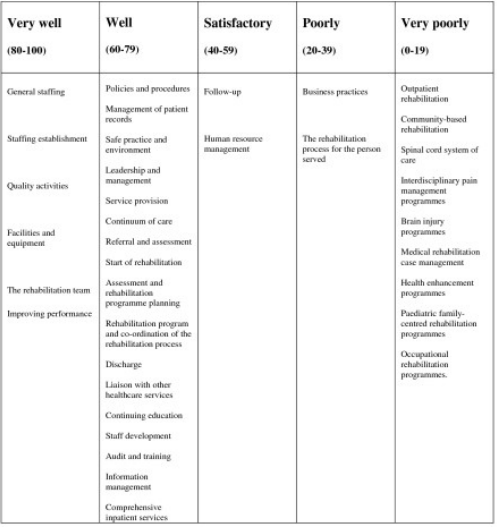
Extent to which issues were covered in the AFRM standards (median VAS score).

**Table 3:** Extent to which issues are covered in the standards.

**VAS **0 = issue not addressed in the standard considered				
100 = issue addressed in the maximum possible detail				
***Issue***	**AFRM** **VAS median (range)**	**BSRM** **VAS median (range)**	**CARF** **VAS median (range)**	**EQUIP** **VAS median** **(range)**

General staffing	90 (76–98)	72 (26–81)	79 (70–94)	37 (18–80)
Staffing establishment	95 (77–98)	58 (25–81)	79 (70–94)	37 (18–80)
Policies and procedures	78 (64–91)	63 (51–81)	79 (70–94)	74 (38–80)
Continuing education	74 (71–74)	63 (41–77)	79 (44–87)	50 (38–80)
Management of patient records	74 (68–79)	62 (12–74)	79 (67–94)	80 (38–95)
Quality activities	95 (92–97)	55 (44–72)	79 (67–94)	90 (65–95)
Facilities and equipment	88 (87–95)	64 (32–68)	69 (50–75)	31 (25–90)
Service provision	79 (79–92)	64 (45–84)	79 (67–92)	50 (37–82)
The rehabilitation team	98 (97–98)	63 (23–79)	79 (71–94)	15 (5–39)
Referral and assessment	65 (57–79)	63 (62–84)	79 (52–94)	60 (27–85)
Start of rehabilitation	65 (58–75)	64 (62–85)	78 (62–94)	14 (10–32)
Assessment and rehabilitation programme planning	65 (59–76)	63 (62–86)	79 (62–94)	15 (10–32)
Rehabilitation programme and co-ordination of the rehabilitation process	65 (59–79)	63 (62–86)	79 (62–94)	16 (10–32)
Discharge	65 (59–80)	70 (63–86)	79 (55–94)	75 (17–87)
Follow-up	59 (15–69)	70 (63–85)	29 (12–68)	73 (18–75)
Staff development/audit and training	78 (76–93)	76 (74–84)	64 (59–75)	72 (21–74)
Liaison with other healthcare services	73 (63–78)	75 (73–84)	27 (20–62)	58 (13–70)
Business practices	25 (15–60)	12 (8–13)	89 (82–95)	45 (20–62)
Rehabilitation process for the person served	25 (15–60)	45 (16–49)	92 (69–95)	30 (20–35)
Comprehensive inpatient rehabilitation programmes	64 (35–72)	66 (48–84)	93 (75–95)	10 (10–23)
Spinal cord system of care	5 (3–6)	4 (4–12)	92 (75–93)	5 (5–10)
Interdisciplinary pain rehabilitation programs	4 (2–5)	4 (1–4)	93 (75–93)	5 (5–10)
Brain injury programs	4 (4–5)	4 (4–6)	93 (75–94)	5 (5–10)
Outpatient medical rehabilitation programmes	14 (11–24)	73 (45–82)	93 (75–93)	7 (6–10)
Home and community-based rehabilitation	14 (13–25)	73 (45–85)	92 (75–93)	7 (6–10)
Medical rehabilitation case management	3 (2–3)	3 (2–6)	93 (75–95)	7 (6–10)
Health enhancement programs	2 (1–2)	1 (1–4)	93 (75–94)	5 (2–10)
Pediatric family-centred rehabilitation programs	1 (1–2)	2 (1–2)	93 (75–94)	5 (2–10)
Occupational rehabilitation programs	2 (1–5)	3 (1–5)	93 (75–95)	7 (4–8)
Continuum of care	79 (60–83)	70 (54–89)	90 (82–91)	95 (47–97)
Leadership and management	73 (68–83)	70 (54–84)	90 (82–93)	95 (47–95)
Human resource management	48 (34–83)	63 (42–72)	90 (85–93)	95 (47–97)
Information management	78 (75–87)	63 (35–68)	90 (68–93)	95 (47–95)
Safe practice and environment	67 (33–72)	8 (6–19)	80 (80–93)	95 (47–97)
Improving performance	96 (62–96)	61 (37–63)	90 (73–93)	95 (47–95)

Of the issues addressed very well (median VAS (Visual Analogue Scale) 80–100) by the AFRM standards, EQUIP was the only other set of standards to receive similarly high scores on some of theses issues, although CARF received a score of greater than 70 for the majority of these issues.

Of the issues well covered (median VAS 60–79) by the AFRM standards, the BSRM standards received a similar score. The CARF standards also received similar scores for these issues, apart from a very low score for liaison with other healthcare services. EQUIP received similar scores for policies and procedures, management of patient records, staff development, referral, assessment and discharge; and lower scores for continuing education, service provision, start of rehabilitation, assessment and program planning, rehabilitation process, liaison with other healthcare services and comprehensive inpatient rehabilitation services. EQUIP received higher scores for continuum of care, leadership and management, information management and safe practice and environment.

Of the issues covered to a satisfactory level (median VAS 40–59) by the AFRM standards, follow-up was covered marginally more thoroughly by the BSRM standards and EQUIP, but considerably less well in the CARF standards. Human resource management were covered well in the CARF and EQUIP standards.

Of the issues covered poorly (median VAS 20–39) in the AFRM standards, business practices were covered slightly more comprehensively in EQUIP, in considerably more detail in the CARF standards and less so in the BSRM standards.

Issues covered very poorly (median VAS 0–19) were also covered to a very limited extent in EQUIP. Outpatient and community-based rehabilitation were addressed to a high level (median VAS score 73) in the BSRM standards, but the other issues were not. All the issues very poorly covered by the AFRM standards were addressed in great detail by the CARF standards.

The AFRM standards covered the following issues to a similar extent to the other rehabilitation service standards assessed:

(1) Policies and procedures, management of patient records

(2) Facilities and equipment

(3) General staffing, staffing establishment, the rehabilitation team

(4) Service provision, referral and assessment, start of rehabilitation, assessment and rehabilitation program planning, rehabilitation program and co-ordination of rehabilitation process, discharge, liaison with other healthcare facilities

(5) Quality activities, improving performance, continuing education, staff development, audit and training

(6) Comprehensive inpatient rehabilitation programs

The next section comments on the relevance of these issues.

### Clinical relevance to a general rehabilitation service

The majority of the issues under consideration in the current analysis were considered highly relevant (median VAS 90+) to a general rehabilitation service. Staff development, human resource management, information management and safe practice and environment were also considered very relevant (median VAS 80+) as were business practices and medical case-management (median VAS 70+). The spinal cord system of care, interdisciplinary pain management rehabilitation programs and brain injury programs (median VAS 24) and health enhancement programmes, paediatric programs and occupational rehabilitation programs (median VAS less than 10) were considered less relevant to general rehabilitation, although they are clearly appropriate for specialised rehabilitation services. The only additional issues suggested for consideration were assessment of clinical outcomes and a community advocacy role. The clinical relevance of the components of the standards is listed in Table 4.

**Table 4:** Clinical relevance to a general rehabilitation service.

**VAS **0 = issue not addressed in the standard considered	
100 = issue addressed in the maximum possible detail	

***Issue***	**VAS median** **(range)**

General staffing	95 (95–97)
Staffing establishment	95 (95–97)
Policies and procedures	95 (89–97)
Continuing education	93 (90–95)
Management of patient records	97 (95–97)
Quality activities	97 (97–97)
Facilities and equipment	97 (91–98)
Service provision	96 (91–97)
The rehabilitation team	97 (97–98)
Referral and assessment	95 (84–97)
Start of rehabilitation	97 (90–98)
Assessment and rehabilitation programme planning	97 (97–98)
Rehabilitation programme and co-ordination of the rehabilitation process	97 (97–98)
Discharge	97 (86–98)
Follow-up	97 (86–98)
Staff development/audit and training	87 (85–97)
Liaison with other healthcare services	98 (97–98)
Business practices	75 (65–87)
Rehabilitation process for the person served	97 (95–97)
Comprehensive inpatient rehabilitation programmes	97 (95–97)
Spinal cord system of care	24 (8–33)
Interdisciplinary pain rehabilitation programs	24 (8–33)
Brain injury programs	24 (8–33)
Outpatient medical rehabilitation programmes	95 (94–97)
Home and community-based rehabilitation	95 (93–97)
Medical rehabilitation case management	70 (34–78)
Health enhancement programs	9 (2–10)
Pediatric family-centred rehabilitation programs	5 (2–9)
Occupational rehabilitation programs	9 (8–31)
Continuum of care	90 (80–95)
Leadership and management	90 (80–95)
Human resource management	88 (80–89)
Information management	88 (74–89)
Safe practice and environment	88 (80–91)
Improving performance	97 (90–98)

### Triangulation

With regards to the repeatability of the VAS in this qualitative analysis, the results are equivocal. For assessment of clinical relevance, the range is small for the majority of issues. Mid-range scores are less consistent. For the assessment of opinion with regard to extent to which issues are addressed in the standards, the VAS range is wide, with mid-range scores again associated with a large variation when the assessment is repeated. Low scores are, however, almost all associated with a very small variation, and may therefore be considered repeatable. Large VAS range variations are particularly noted in the assessment of the extent to which issues are addressed in the EQuIP standards. This may reflect the fact that the EQuIP standards are conceptual rather than directive in nature, and may therefore be subject to a different interpretation under different circumstances.

The use of VAS as an assessment tool was compared with another qualitative analysis method (see Table 5). The BSRM standards were considered to address the issues of continuum of care, leadership and management, human resource management, information management, safe practice and environment and improving performance to a lesser extent than EQUIP. This is in agreement with the median VAS scores obtained for these issues (95 in all cases for EQUIP and 8–70 for the BSRM standards). CARF was considered to have covered these issues to an equivalent extent when compared with EQUIP. The median VAS score of 80 for safe practice and environment and 90 for all the other issues are equivalent to the scores for the EQUIP standards. There is therefore convergence between the findings of these two methods of assessing the standards.

**Table 5:** Sample of alternative method of analysis (baseline = EQuIP).

**ISSUE**	**BSRM**	**CARF**
Continuum of care	L	E
Leadership and management	L	E
Human resource management	NA	E
Information management	L	E
Safe practice and environment	L	E
Improving performance	L	E

D = issue addressed in greater detail than in the baseline standard

E = equivalent

L = less detail

## Discussion

The October 2003 draft AFRM rehabilitation service standards are consistent, concise, provide definitions, and cover the majority of clinically relevant issues to an extent similar to the other available rehabilitation service standards. Service evaluation methods are provided via advice to utilise EQuIP for the purposes of quality assurance. In a direct sense, the AFRM standards contain the EQuiP standards.

The descriptive analysis suggested that the BSRM standards were developed in the most rigorous way, but may have limited face validity due to the lack of use in the field. Due to their widespread utilisation in the field, the CARF and EQUIP standards may be considered valid despite a less rigorous approach to their development. The AFRM standards were developed in a less rigorous way than the BSRM standards, but are more widely utilised.

The results of the thematic analysis suggested that the BSRM standards lack definitions and evaluation methods, that the CARF standards may be considered over-inclusive, and that the EQUIP standards are not sufficiently specific to rehabilitation services. The overall structure of the AFRM standards may therefore be considered the most appropriate compromise as it includes definitions, and is concise and consistent in structure. The AFRM standards suggest the use of the EQUIP standards for use in quality assurance, so may be considered, at least in part, to include an explicit evaluation method.

Content analysis showed that the AFRM standards covered the majority of issues to a similar extent to the other rehabilitation service standards assessed.

The EQUIP standards cover continuum of care, leadership and management, information management and safe practice and environment in more detail than the AFRM standards, and it is therefore appropriate for the use of the EQUIP standards to be recommended in the AFRM standards.

Follow-up and human resource management were covered less well in the AFRM standards and it would be of benefit for these issues to be considered, possibly with reference to the BSRM and EQUIP standards with regard to follow-up, and with reference to the CARF and EQUIP standards with regard to human resource management.

Of the issues covered poorly by the AFRM standards, the rehabilitation process for the person served was considered highly clinically relevant, and should therefore be considered for inclusion in revised standards, possibly with reference to the CARF standards which address this issue well. Outpatient and community-based rehabilitation were also considered clinically relevant, and their inclusion should also be considered with reference to the BSRM and CARF standards where they are well covered. Business practices was considered slightly less clinically relevant, but should still be considered for inclusion in Australian-based standards, as private practice is becoming an increasingly important aspect of medical practice in Australia [11]. Spinal cord system of care, interdisciplinary pain management programs, brain injury programs, medical rehabilitation case management, health enhancement programs, paediatric family-centred rehabilitation programs and occupational rehabilitation programs were not considered highly clinically relevant to a general rehabilitation service. However, separate sub-specialty standards may be of use, and reference to the CARF standards may be of benefit in their development, in addition to published sub-specialty standards [12,13].

Triangulation via repetition of the assessment and comparison with an alternative methodology suggested that VAS may be an appropriate way of assessing opinion. Further work is required to assess this methodology, including repetition of the analysis between raters and across time.

It would also be of benefit to improve the scientific validity of the AFRM standards via the use of formal methods of service standard development and the publication of this process in peer-reviewed journals.

The current analysis therefore suggests that the AFRM rehabilitation service standards are of high quality but could be improved by the inclusion of additional sections with reference to the BSRM and CARF standards, followed by formal evaluation and formal field testing. Since completion of this project the AFRM rehabilitation standards have been up-dated [14] using information from a number of sources including the AFRM Special Projects Committee which had access to the preliminary results of this study.

## Conclusion

This qualitative analysis compared AFRM, BSRM and CARF rehabilitation service standards. The use of VAS demonstrated reliability and validity in this qualitative analysis. AFRM standards were found to be consistent and concise, to provide definitions and to cover the majority of clinically relevant issues to an extent similar to other rehabilitation service standards. Inclusion of standards for business practices, rehabilitation process for the person served, and outpatient and community-based rehabilitation services should be considered by the AFRM.

This article outlines a recently developed, reliable and valid method of evaluating service standards. The AFRM standards were found to compare well with other standards available in Australia and overseas. They should therefore be considered a suitable framework for assessing rehabilitation services and providing advice regarding the rehabilitation health workforce. It will be important for the standards to be periodically re-evaluated, field tested and up-dated.

## Methods

The AFRM, BSRM and CARF rehabilitation standards were compared via a published qualitative analysis structure. This dynamic analysis process, suggested by Gifford [15], involves description, classification and connection (see Box 2). The content analysis outlined below was carried out by one rater.

**Figure 2:**
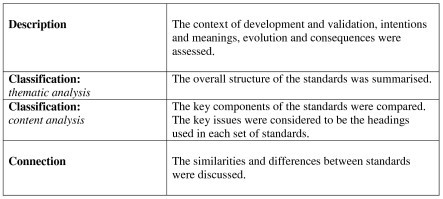
Qualitative analysis structure.

A list was made of the issues covered by the three sets of standards. The degree to which each issue was addressed by the standards was assessed. A Visual Analogue Scale (VAS) was selected as the scoring method for the analysis as it provides a summary measure and is widely used in research and clinical practice. The standards were compared and the clinical relevance of the issues assessed using a piloted VAS [see Additional file 1]. A list was made of any additional areas the investigator felt should have been covered by the standards. The analysis was repeated on three occasions at least one week apart.

A comparison of the standards had been carried out several months previously using an alternative methodology which involved stating whether components of the standards were covered in equivalent, more or less detail.

Triangulation was carried out via comparison of the VAS scores obtained on the three separate occasions, comparison with the results of the alternative method of analysis, assessment of clinical relevance and consideration of any additional areas that should have been covered by the standards.

## Competing interests

During the preparation of this article, the first author accepted an invitation to participate in the AFRM Special Projects Committee. This committee is responsible for writing and up-dating the AFRM rehabilitation service standards.

## Authors' contributions

SKG designed the study, carried out the analysis and drafted the manuscript, IDC assisted in the design of the study and helped draft the manuscript, HGD provided advice regarding the initial study concepts and helped draft the manuscript. All authors read and approved the final manuscript.

## Supplementary Material

Additional file 1 Standards comparison proforma. The proforma used in the study Click here for file
