# Supplementary material for: Analysis of draft Australian rehabilitation service standards: comparison with international standards
Source: Aust New Zealand Health Policy. 2008 Jun 30;5:15. doi: 10.1186/1743-8462-5-15 (PMC2474639; doi:10.1186/1743-8462-5-15)
Supplement: Additional file 1 — Standards comparison proforma. The proforma used in the study [file 1743-8462-5-15-S1.doc]

#### **Appendix 1: Content analysis proforma**

#### A comparison of AFRM, BSRM and CARF standards

#### Content analysis

#### Please complete the visual analogue scales (VAS) by placing an “x” on the lines in the attached tables.

For example, if you feel that AFRM addresses the issue of general staffing in almost the maximum possible detail place an “x” as shown below:

| Extent to which issues are addressed in AFRM | |
| --- | --- |
| Issue | **0=not addressed**  **100=addressed in maximum possible detail** |
| General staffing | 0 100  [ ] |
| Leadership and management | 0 100  [ ] |

#### Thankyou

#### Susan Rundle

(susanrundle2@ausdoctors.net)

| Extent to which issues are addressed in AFRM standards | |
| --- | --- |
| Issue | **0=not addressed**  **100=addressed in maximum possible detail** |
| General staffing | 0 100  [ ] |
| Staffing establishment | 0 100  [ ] |
| Policies and procedures | 0 100  [ ] |
| Continuing education | 0 100  [ ] |
| Management of patient records | 0 100  [ ] |
| Quality activities | 0 100  [ ] |
| Facilities and equipment | 0 100  [ ] |
| Service provision | 0 100  [ ] |
| The rehabilitation team | 0 100  [ ] |
| Referral and assessment | 0 100  [ ] |
| Start of rehabilitation | 0 100  [ ] |
| Assessment and rehabilitation programme planning | 0 100  [ ] |
| Rehabilitation programme and co-ordination of the rehabilitation process | 0 100  [ ] |
| Discharge | 0 100  [ ] |
| Follow-up | 0 100  [ ] |

| Extent to which issues are addressed in AFRM standards (cont) | |
| --- | --- |
| Issue | **0=not addressed**  **100=addressed in maximum possible detail** |
| Staff development / audit and training | 0 100  [ ] |
| Liaison with other healthcare services | 0 100  [ ] |
| Business practices | 0 100  [ ] |
| Rehabilitation process for the person served | 0 100  [ ] |
| Comprehensive inpatient rehabilitation programmes | 0 100  [ ] |
| Spinal cord system of care | 0 100  [ ] |
| Interdisciplinary pain rehabilitation programs | 0 100  [ ] |
| Brain injury programs | 0 100  [ ] |
| Outpatient medical rehabilitation programmes | 0 100  [ ] |
| Home and community-based rehabilitation | 0 100  [ ] |
| Medical rehabilitation case management | 0 100  [ ] |
| Health enhancement programs | 0 100  [ ] |
| Pediatric family-centred rehabilitation programs | 0 100  [ ] |
| Occupational rehabilitation programs | 0 100  [ ] |

| Extent to which issues are addressed in AFRM standards (cont) | |
| --- | --- |
| Continuum of care | 0 100  [ ] |
| Leadership and management | 0 100  [ ] |
| Human resource management | 0 100  [ ] |
| Information management | 0 100  [ ] |
| Safe practice and environment | 0 100  [ ] |
| Improving performance | 0 100  [ ] |

| **Comments on AFRM standards** |
| --- |
|  |

| Extent to which issues are addressed in BSRM standards | |
| --- | --- |
| Issue | **0=not addressed**  **100=addressed in maximum possible detail** |
| General staffing | 0 100  [ ] |
| Staffing establishment | 0 100  [ ] |
| Policies and procedures | 0 100  [ ] |
| Continuing education | 0 100  [ ] |
| Management of patient records | 0 100  [ ] |
| Quality activities | 0 100  [ ] |
| Facilities and equipment | 0 100  [ ] |
| Service provision | 0 100  [ ] |
| The rehabilitation team | 0 100  [ ] |
| Referral and assessment | 0 100  [ ] |
| Start of rehabilitation | 0 100  [ ] |
| Assessment and rehabilitation programme planning | 0 100  [ ] |
| Rehabilitation programme and co-ordination of the rehabilitation process | 0 100  [ ] |
| Discharge | 0 100  [ ] |
| Follow-up | 0 100  [ ] |

| Extent to which issues are addressed in BSRM standards (cont) | |
| --- | --- |
| Issue | **0=not addressed**  **100=addressed in maximum possible detail** |
| Staff development / audit and training | 0 100  [ ] |
| Liaison with other healthcare services | 0 100  [ ] |
| Business practices | 0 100  [ ] |
| Rehabilitation process for the person served | 0 100  [ ] |
| Comprehensive inpatient rehabilitation programmes | 0 100  [ ] |
| Spinal cord system of care | 0 100  [ ] |
| Interdisciplinary pain rehabilitation programs | 0 100  [ ] |
| Brain injury programs | 0 100  [ ] |
| Outpatient medical rehabilitation programmes | 0 100  [ ] |
| Home and community-based rehabilitation | 0 100  [ ] |
| Medical rehabilitation case management | 0 100  [ ] |
| Health enhancement programs | 0 100  [ ] |
| Pediatric family-centred rehabilitation programs | 0 100  [ ] |
| Occupational rehabilitation programs | 0 100  [ ] |

| Extent to which issues are addressed in BSRM standards (cont) | |
| --- | --- |
| Continuum of care | 0 100  [ ] |
| Leadership and management | 0 100  [ ] |
| Human resource management | 0 100  [ ] |
| Information management | 0 100  [ ] |
| Safe practice and environment | 0 100  [ ] |
| Improving performance | 0 100  [ ] |

| **Comments on BSRM standards** |
| --- |
|  |

| Extent to which issues are addressed in CARF standards | |
| --- | --- |
| Issue | **0=not addressed**  **100=addressed in maximum possible detail** |
| General staffing | 0 100  [ ] |
| Staffing establishment | 0 100  [ ] |
| Policies and procedures | 0 100  [ ] |
| Continuing education | 0 100  [ ] |
| Management of patient records | 0 100  [ ] |
| Quality activities | 0 100  [ ] |
| Facilities and equipment | 0 100  [ ] |
| Service provision | 0 100  [ ] |
| The rehabilitation team | 0 100  [ ] |
| Referral and assessment | 0 100  [ ] |
| Start of rehabilitation | 0 100  [ ] |
| Assessment and rehabilitation programme planning | 0 100  [ ] |
| Rehabilitation programme and co-ordination of the rehabilitation process | 0 100  [ ] |
| Discharge | 0 100  [ ] |
| Follow-up | 0 100  [ ] |

| Extent to which issues are addressed in CARF standards (cont) | |
| --- | --- |
| Issue | **0=not addressed**  **100=addressed in maximum possible detail** |
| Staff development / audit and training | 0 100  [ ] |
| Liaison with other healthcare services | 0 100  [ ] |
| Business practices | 0 100  [ ] |
| Rehabilitation process for the person served | 0 100  [ ] |
| Comprehensive inpatient rehabilitation programmes | 0 100  [ ] |
| Spinal cord system of care | 0 100  [ ] |
| Interdisciplinary pain rehabilitation programs | 0 100  [ ] |
| Brain injury programs | 0 100  [ ] |
| Outpatient medical rehabilitation programmes | 0 100  [ ] |
| Home and community-based rehabilitation | 0 100  [ ] |
| Medical rehabilitation case management | 0 100  [ ] |
| Health enhancement programs | 0 100  [ ] |
| Pediatric family-centred rehabilitation programs | 0 100  [ ] |
| Occupational rehabilitation programs | 0 100  [ ] |

| Extent to which issues are addressed in CARF standards (cont) | |
| --- | --- |
| Continuum of care | 0 100  [ ] |
| Leadership and management | 0 100  [ ] |
| Human resource management | 0 100  [ ] |
| Information management | 0 100  [ ] |
| Safe practice and environment | 0 100  [ ] |
| Improving performance | 0 100  [ ] |

| **Comments on CARF standards** |
| --- |
|  |

| Extent to which issues are addressed in EQUIP standards | |
| --- | --- |
| Issue | **0=not addressed**  **100=addressed in maximum possible detail** |
| General staffing | 0 100  [ ] |
| Staffing establishment | 0 100  [ ] |
| Policies and procedures | 0 100  [ ] |
| Continuing education | 0 100  [ ] |
| Management of patient records | 0 100  [ ] |
| Quality activities | 0 100  [ ] |
| Facilities and equipment | 0 100  [ ] |
| Service provision | 0 100  [ ] |
| The rehabilitation team | 0 100  [ ] |
| Referral and assessment | 0 100  [ ] |
| Start of rehabilitation | 0 100  [ ] |
| Assessment and rehabilitation programme planning | 0 100  [ ] |
| Rehabilitation programme and co-ordination of the rehabilitation process | 0 100  [ ] |
| Discharge | 0 100  [ ] |
| Follow-up | 0 100  [ ] |

| Extent to which issues are addressed in EQUIP standards (cont) | |
| --- | --- |
| Issue | **0=not addressed**  **100=addressed in maximum possible detail** |
| Staff development / audit and training | 0 100  [ ] |
| Liaison with other healthcare services | 0 100  [ ] |
| Business practices | 0 100  [ ] |
| Rehabilitation process for the person served | 0 100  [ ] |
| Comprehensive inpatient rehabilitation programmes | 0 100  [ ] |
| Spinal cord system of care | 0 100  [ ] |
| Interdisciplinary pain rehabilitation programs | 0 100  [ ] |
| Brain injury programs | 0 100  [ ] |
| Outpatient medical rehabilitation programmes | 0 100  [ ] |
| Home and community-based rehabilitation | 0 100  [ ] |
| Medical rehabilitation case management | 0 100  [ ] |
| Health enhancement programs | 0 100  [ ] |
| Pediatric family-centred rehabilitation programs | 0 100  [ ] |
| Occupational rehabilitation programs | 0 100  [ ] |

| Extent to which issues are addressed in EQUIP standards (cont) | |
| --- | --- |
| Continuum of care | 0 100  [ ] |
| Leadership and management | 0 100  [ ] |
| Human resource management | 0 100  [ ] |
| Information management | 0 100  [ ] |
| Safe practice and environment | 0 100  [ ] |
| Improving performance | 0 100  [ ] |

| **Comments on EQUIP standards** |
| --- |
|  |

| Clinical relevance to a general rehabilitation service | |
| --- | --- |
| Issue | **0=not clinically relevant**  **100=maximal clinical relevance** |
| General staffing | 0 100  [ ] |
| Staffing establishment | 0 100  [ ] |
| Policies and procedures | 0 100  [ ] |
| Continuing education | 0 100  [ ] |
| Management of patient records | 0 100  [ ] |
| Quality activities | 0 100  [ ] |
| Facilities and equipment | 0 100  [ ] |
| Service provision | 0 100  [ ] |
| The rehabilitation team | 0 100  [ ] |
| Referral and assessment | 0 100  [ ] |
| Start of rehabilitation | 0 100  [ ] |
| Assessment and rehabilitation programme planning | 0 100  [ ] |
| Rehabilitation programme and co-ordination of the rehabilitation process | 0 100  [ ] |
| Discharge | 0 100  [ ] |
| Follow-up | 0 100  [ ] |

| Clinical relevance to a general rehabilitation service (cont) | |
| --- | --- |
| Issue | **0=not addressed**  **100=addressed in maximum possible detail** |
| Staff development / audit and training | 0 100  [ ] |
| Liaison with other healthcare services | 0 100  [ ] |
| Business practices | 0 100  [ ] |
| Rehabilitation process for the person served | 0 100  [ ] |
| Comprehensive inpatient rehabilitation programmes | 0 100  [ ] |
| Spinal cord system of care | 0 100  [ ] |
| Interdisciplinary pain rehabilitation programs | 0 100  [ ] |
| Brain injury programs | 0 100  [ ] |
| Outpatient medical rehabilitation programmes | 0 100  [ ] |
| Home and community-based rehabilitation | 0 100  [ ] |
| Medical rehabilitation case management | 0 100  [ ] |
| Health enhancement programs | 0 100  [ ] |
| Pediatric family-centred rehabilitation programs | 0 100  [ ] |
| Occupational rehabilitation programs | 0 100  [ ] |

| Clinical relevance to a general rehabilitation service (cont) | |
| --- | --- |
| Continuum of care | 0 100  [ ] |
| Leadership and management | 0 100  [ ] |
| Human resource management | 0 100  [ ] |
| Information management | 0 100  [ ] |
| Safe practice and environment | 0 100  [ ] |
| Improving performance | 0 100  [ ] |

| Additional issues | **Clinical relevance**  **0=not clinically relevant**  **100=maximal clinical relevance** |
| --- | --- |
|  | 0 100  [ ] |
|  | 0 100  [ ] |
|  | 0 100  [ ] |
|  | 0 100  [ ] |
|  | 0 100  [ ] |
|  | 0 100  [ ] |
|  | 0 100  [ ] |

| **Comments on clinical relevance** |
| --- |
|  |
